# CXCR2 intrinsically drives the maturation and function of neutrophils in mice

**DOI:** 10.3389/fimmu.2022.1005551

**Published:** 2022-10-13

**Authors:** Pauline Delobel, Benjamin Ginter, Eliane Rubio, Karl Balabanian, Gwendal Lazennec

**Affiliations:** ^1^ CNRS, UMR9005, Sys2Diag-ALCEN, Cap delta, Montpellier, France; ^2^ CNRS, GDR 3697 “Microenvironment of tumor niches”, Micronit, France; ^3^ Université Paris-Cité, Institut de Recherche Saint-Louis, INSERM U1160, Paris, France

**Keywords:** chemokine receptors, Cxcr2, neutrophils, inflammation, tumor microenvironment

## Abstract

Neutrophils play a major role in the protection from infections but also in inflammation related to tumor microenvironment. However, cell-extrinsic and -intrinsic cues driving their function at steady state is still fragmentary. Using *Cxcr2* knock-out mice, we have evaluated the function of the chemokine receptor Cxcr2 in neutrophil physiology. We show here that Cxcr2 deficiency decreases the percentage of mature neutrophils in the spleen, but not in the bone marrow (BM). There is also an increase of aged CD62L^lo^ CXCR4^hi^ neutrophils in the spleen of KO animals. Spleen *Cxcr2^-/-^* neutrophils display a reduced phagocytic ability, whereas BM neutrophils show an enhanced phagocytic ability compared to WT neutrophils. Spleen *Cxcr2^-/-^* neutrophils show reduced reactive oxygen species production, F-actin and α-tubulin levels. Moreover, spleen *Cxcr2^-/-^* neutrophils display an altered signaling with reduced phosphorylation of ERK1/2 and p38 MAPK, impaired PI3K-AKT, NF-κB, TGFβ and IFNγ pathways. Altogether, these results suggest that Cxcr2 is essential for neutrophil physiology.

## Introduction

One of the first line of defense against pathogens such as bacteria, fungi, or parasites involves neutrophils, which are key mediator of innate immunity and inflammation. Neutrophils use different ways to clear the infection, including bacterial uptake (phagocytosis), phagolysosomal degradation of bacteria with a cocktail of antimicrobial factors and reactive oxygen species (ROS) (oxidative burst) or release of granules to neutralize extracellular pathogens (degranulation) (1). In addition, when intruders are too large or have escaped the other microbial killing processes, neutrophils can extrude a physical barrier to pathogen dissemination, called a neutrophil extracellular trap (NET), containing DNA, histones and granule proteases (2).

Neutrophils are the most abundant circulating leukocytes, representing 50% to 70% of all circulating leukocytes in humans and about 10 to 25% in mice (3). Neutrophils are relatively short lived cells (4), even if recent studies have questioned this aspect and it is now believed that human and murine neutrophils could have a half-life of 5 days or 18h, respectively (5).

Neutrophils arise from granulocyte–monocyte progenitors (GMPs), mostly within the bone marrow (BM) during hematopoiesis in response to several cytokines, principally granulocyte colony–stimulating factor (G-CSF) (6), but also from extramedullary tissues such as spleen (7–9). The first progenitor that is ‘neutrophil committed’ is the neutrophil promyelocyte (10), which then maturates through granulocyte-committed precursors comprising myeloblasts, promeylocytes and myelocytes, to a post-mitotic or transition pool of metamyelocytes, band cells and segmented neutrophils (11). Mature post-mitotic neutrophils are released from the BM into the peripheral blood, extravasate from circulation into the tissues under the coordinate regulation of various adhesion molecules and chemokines (12). They are involved not only in the control of inflammation following infections or injuries but have also pro or anti-tumoral actions as tumor associated neutrophils (TANs) (13–15). In inflammatory sites, neutrophils fight the injury or infection and undergo apoptosis and are phagocyted by macrophages, once inflammation has been resolved (16). Senescent neutrophils can also home back to the BM in a Cxcr4-dependent mechanism (17). The terminal differentiation of neutrophils in the BM, before release into the bloodstream, is still a subject of debate, as some steps of maturation could occur in other organs such as the spleen (9) and in the same line, neutrophil progenitors have been found in the spleen (18). Maturation markers of neutrophils are also currently discussed, but classically Ly6G, Cd101, Cxcr4 and Cxcr2 are used in mice (8, 12).

Cxcr2 appears as one of the key chemokine receptor expressed by neutrophils both in mice and humans (19, 20). Cxcr2 binds the chemokines Cxcl1, 2, 3, 5, 6, 7 and 8 in human, which all have pro-angiogenic properties and are located in a short cluster of chromosome 4 (21–23). Cxcr2 ligand action is conditioned by its interaction with proteoglycans (24) and Cxcr2 signals through multiple pathways including PI3K and Src (25). In addition, recent work has highlighted the role of Cxcr2 in tumorigenesis, in particular through tumor-associated neutrophils (14, 23, 26, 27), but also in the effects microbiota on pituitary function (28). *Cxcr2* knockout mice have been generated and are characterized by a splenomegaly due to an increase of metamyelocytes, mature neutrophils and B lymphocytes (29). These mice also display a defect in neutrophil recruitment after infection (30).

So far, the mechanism of Cxcr2 action in neutrophils remains poorly understood. In this study, we have investigated the role of Cxcr2 in mouse neutrophils in the spleen and the BM, taking advantage of *Cxcr2^-/-^* mice. Our data show that Cxcr2 impairment in neutrophils affects differently spleen and BM neutrophils in terms of maturation, phagocytosis and ROS production. Moreover, we analyzed at the transcriptomic level the pathways that were altered by *Cxcr2* deletion in neutrophils. Altogether, these results suggest that proper Cxcr2 expression and function is required for maturation and effector functions of peripheral neutrophils.

## Materials and methods

### Animal models and housing

All animal experiments conformed to our animal protocols that were reviewed and approved by the Institutional Animal Care and Use Committee. *Cxcr2^-/-^* mice (29) were obtained from the Jackson Laboratory. *Cxcr2^-/-^* mice were backcrossed in FVB genetic background for more than 12 generations. Control (WT) mice were also in a FVB background. All mice were housed in a SOPF (Specific and Opportunistic Pathogen Free) animal facility.

### Isolation of cells

Cells from the BM were isolated by centrifugation from the femurs and tibias of the animals, whereas spleens were mashed on 100 µm nylon cell strainer. After centrifugation, red blood cells were eliminated by treatment with ACK buffer (0.155 mM NH4Cl, 1 mM KHCO3, 0.1 mM EDTA) and filtered on a 40 µm nylon cell strainer. After ACK treatment, cells were filtered on a 40 µm nylon cell strainer. For neutrophil isolation, a first enrichment with EasySep™ Mouse CD11b Positive Selection kit (StemCell technologies, Grenoble, France) was performed followed by cell sorting of CD45+ CD11b+ Ly6G+ F4/80- cells on an ARIA IIu FACS sorter (Becton Dickinson, Le Pont-de-Claix, France).

### Flow cytometry

Flow cytometry experiments were performed with the following conjugated antibodies from Biolegend (London, United Kingdom): anti-mouse CD11b (clone M1/70), CD45 (clone 30-F11), Cxcr2 (clone SA044G4), Ly6G (clone 1A8), CD62L (clone MEL-14) or BD Biosciences (BD Biosciences, Le Pont-de-Claix, France): Cxcr4 (clone 2B11), or Ebiosciences (Fisher Scientific, Illkirch, France): CD101 (clone Moushi101), Fixable viability dye (65-0866). Flow analysis was performed on live singlets with a LSR II Fortessa flow cytometer (Becton Dickinson, Le Pont-de-Claix, France). Data were analyzed using FlowJo (Tree Star).

### Intracellular flow cytometry

Cells were first stained with antibodies directed against extracellular markers (CD45, CD11b, Ly6G) and then permeabilized with Cytofix/Cytoperm and Permwash buffer (BD Biosciences). Intracellular staining of F-actin was performed with Phalloidin, Fluorescein Isothiocyanate Labeled (Sigma-Aldrich, ref P5282), whereas α-tubulin was labeled with α-Tubulin antibody (clone 11H10, Cell Signaling, ref 2125) followed by secondary an anti-rabbit IgG antibody coupled to Alexa Fluor 555 (ThermoFisher, ref A27039). Phospho-p42/44 MAPK ERK1/2 (Thr 202/Tyr 204) (Cell signaling, ref 4370) followed by secondary an anti-rabbit IgG antibody coupled to Alexa Fluor 555 (ThermoFisher, ref A27039) was used to detect Phospho-p42/44 MAPK ERK1/2. p-p38 MAPK (Thr 180/Tyr 182) coupled to PE (cell Signaling 6908), was used to detect phospho-p38 MAPK.

### Annexin V staining and measure of mortality

To determine the proportion of apoptotic and dead cells, fresh neutrophils were stained with Annexin-FITC and propidium iodide (PI) according to manufacturer instruction (Invitrogen, ref V13242).

### Phagocytosis assay

CD45+ CD11b+ Ly6G+ neutrophils were incubated for various times at 37°C with E. coli Red Phrodo bioparticles (ThermoFisher, Illkirch, France) at a concentration of 15 µg/ml and analyzed in a kinetic manner by flow cytometry with a LSR II Fortessa flow cytometer (Becton Dickinson, Le Pont-de-Claix, France). Data were analyzed using FlowJo (Tree Star). When opsonized particles were used, they were opsonized for 1h at 37°C with opsonizing reagent (E2870, ThermoFisher, Illkirch, France), according to manufacturer instructions.

### Reactive oxygen species and mitochondrial superoxide quantification

Reactive oxygen species and mitochondrial quantification was performed by labelling fresh CD45+ CD11b+ Ly6G+ neutrophils for 20 min at 37°C, using CellRox Orange reagent (ref. C10443) and Mitosox reagent (ref. M36008) respectively, following the manufacturer’s instructions (Molecular Probes, ThermoFisher Scientific).

### RNA extraction and RNA-seq data processing

Total RNA was isolated using TRIzol reagent (Fisher Scientific, Illkirch, France), as described by the manufacturer. RNA integrity and quality were verified using RNA ScreenTape kit and Tapestation 2200 apparatus from AGILENT (Les Ulis, France). cDNA libraries were synthesized using NEBNext^®^ rRNA Depletion and Ultra™ II Directional RNA Library Prep Kit (New England Biolabs, Evry-Courcouronnes, France). Library quality was checked on Tapestation 2200 apparatus from AGILENT (Les Ulis, France) with DNA 1000 ScreenTape. Samples were sequenced on Novaseq 6000 (Illumina) with an average sequencing depth of 30 million of paired-end reads. Length of the reads was 150 bp. Each 24 Plex Samples was sequenced on one Illumina SP FlowCell (2*800 million of 150bases reads). Raw sequencing data was quality-controlled with the FastQC program. Low quality reads were trimmed or removed using Trimmer (minimum length: 120 bp). Reads were aligned to the mouse reference genome (mm10 build) with the Star tool. Gene counts were obtained by read counting software Htseq. Normalization and differential analysis were performed with the DESeq2 package with Benjamini-Hochberg FDR multiple testing correction (p < 0.05; 1.5-fold or higher change) comparing WT and KO animals. The data discussed in this publication have been deposited in NCBI’s Gene Expression Omnibus (31) and are accessible through GEO Series accession number GSE209860 (https://www.ncbi.nlm.nih.gov/geo/query/acc.cgi?acc=GSE209860).

### Bioinformatic analysis

To assess biological interpretation of the most differentially expressed genes, we used Gene ontology (GO) enrichment analysis. A gene set enrichment analysis (GSEA) was performed using signatures from GSEA collections. A normalized enrichment score (NES) was calculated for each gene set and only gene sets with an adjusted p value < 0.05 were selected.

### Statistics

Statistical analyses were carried out using unpaired Mann-Whitney test.

## Results

### *Cxcr2* invalidation affects the maturation of neutrophils

**Figure 1:**
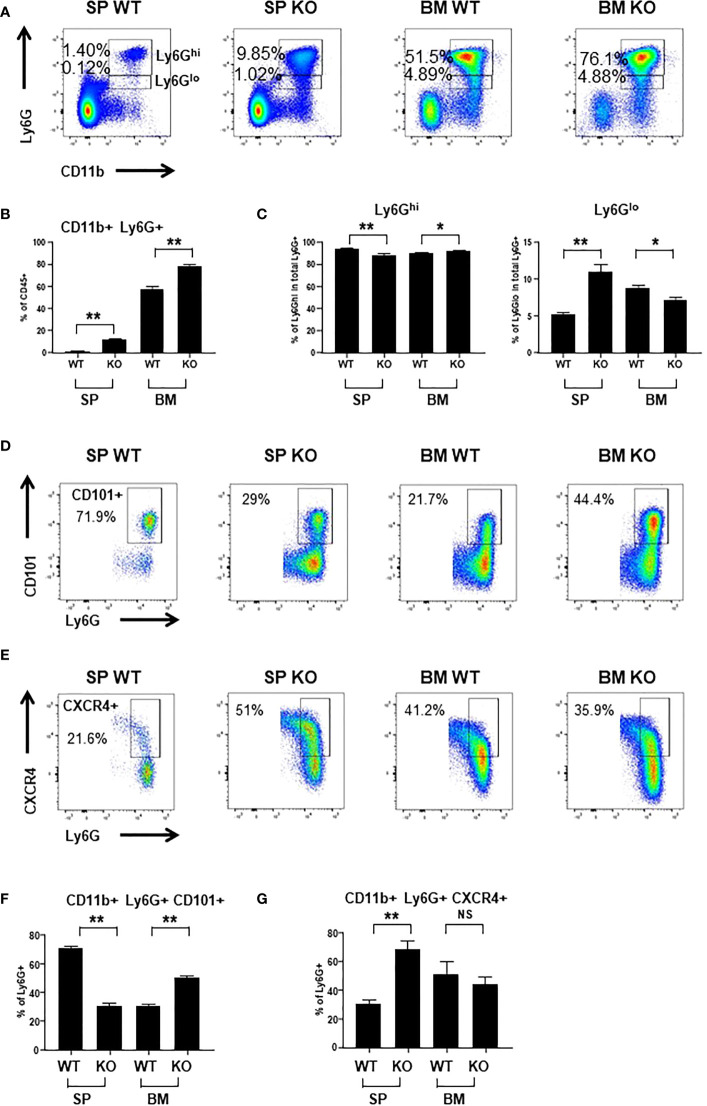
*Cxcr2* knock-out decreases the percentage of mature neutrophils in the spleen. **(A)** Representative dot plots of the gating strategy of CD45+ CD11b+ Ly6G^hi^ and CD45+ CD11b+ Ly6G^lo^ neutrophils among CD45+ cells in WT and *Cxcr2-/-* bone marrow (BM) and spleen (SP). **(B)** Quantification of the percentage of the total neutrophils (CD11b+ Ly6G+: sum of Ly6G^hi^ and Ly6G^lo^) in the CD45+ fraction. **(C)** Quantification of CD11b+ Ly6G^hi^ cells (left panel) and CD11b Ly6G^lo^ (right panel) in the CD11b+ Ly6G+ (Ly6G^hi^ and Ly6G^lo^ fractions) population. **(D)** Gating strategy to identify CD101+ neutrophils in the CD45+ CD11b+ Ly6G+ fraction. **(E)** Gating strategy to identify CXCR4+ neutrophils in the CD45+ CD11b+ Ly6G+ fraction. **(F)** Percentage of mature CD101+ neutrophils in the CD11b+ Ly6G+ fraction. **(G)** Percentage of CXCR4+ neutrophils in the CD11b+ Ly6G+ fraction. Data represent the mean ± SEM of at least 6 animals (Mann-Whitney test, NS: non-significant, *p < 0.05, **p < 0.01).

To evaluate the impact of *Cxcr2* knockout on neutrophil distribution and function, we first measured the presence of CD45+ CD11b+ Ly6G+ neutrophils in the BM and the spleen by flow cytometry (**Figure 1A**). Ly6G+ neutrophils were composed of two subpopulations with high (Ly6G^hi^) or low (Ly6G^lo^) levels of Ly6 (**Figure 1A**). In BM, the percentage of total Ly6G+ (Ly6G^hi^ + Ly6G^lo^) neutrophils was increased by about 30% in *Cxcr2-/-* compared to WT animals, and this was much more pronounced in the spleen, with more than 8 - fold more neutrophils in *Cxcr2-*/- animals (**Figure 1B**). Of particular note, the percentage of CD45+ cells was not different in WT and KO spleens, whereas it slightly increased (88.7% vs 95.2%) in KO BM compared to WT BM (**Supplemental Figure 1**). No major change in FSC and SSC distribution was observed for Ly6G+ neutrophils (**Supplemental Figure 2**). In terms of absolute numbers, we observed a 5-fold increase of the total number of CD45+ cells and CD45+ CD11b+ Ly6G+ neutrophils in the spleen of Cxcr2-/- animals, whereas minimal differences between and Cxcr2-/- and WT animals was seen in BM (no change of the number of CD45+ cells and less than 30% increase for CD45+ CD11b+ Ly6G+) (**Supplemental Figure 3**). This obviously creates a bias in the analysis of any type of subpopulation and for this reason, we decided to continue to look at percentage of subpopulations of neutrophils among neutrophils, to avoid this.

The level of Ly6G expression has been correlated with the degree of maturation of neutrophils with Ly6G^hi^ neutrophils being the most mature (32). In BM, there was a slight reduction of the percentage of immature Ly6G^lo^ neutrophils among total Ly6G+ in *Cxcr2-/-* animals, whereas there was an increase by 2-fold of immature Ly6G^lo^ neutrophils in *Cxcr2-/-* spleen (**Figure 1C**). To strengthen these results, we used the CD101 marker, which characterizes mature neutrophils (8). Among neutrophils, we observed a reduction of CD101+ mature neutrophils in the spleen, but an increase of mature neutrophils in BM (**Figures 1D, F**). Next, we also looked at Cxcr4 expression in neutrophils, which is correlated not only to immaturity of neutrophils (8), but also to aged neutrophils (33). The percentage of Cxcr4+ neutrophils increased twice in the spleen, but was unaffected in the BM (**Figures 1E, G**), confirming the increase of the proportion of immature neutrophils in the neutrophil fraction of the spleen. To assess more precisely whether these neutrophils corresponded to immature or aged neutrophils in the spleen of KO animals, we used the marker CD62L (L-Selectin), in addition to CXCR4 to identify “aged” CD62L^lo^- CXCR4^hi^ neutrophils, as previously described (33). We observed that the proportion of aged CD62L^lo^- CXCR4^hi^ cells among neutrophils increased in KO spleen compared to WT, but there was no significant change in BM (**Supplemental Figure 4**).

To further assess the nature of neutrophils, we isolated by cell sorting CD45+ CD11b+ Ly6G+ neutrophils from WT and KO BM and spleen and colored them with Giemsa (**Supplemental Figure 9**). WT BM neutrophils displayed a condensed round nucleus, with a small cytoplasm, whereas KO BM neutrophils seem to have a larger ring-shaped nucleus, which could suggest that they are more mature. In the spleen, WT neutrophils had a strongly colored ring-shaped nucleus. Spleen KO neutrophils exhibited a larger nucleus with a less pronounced staining and also a cytoplasmic center, which could be reminiscent of band neutrophils (34). These morphological differences suggest that WT and KO neutrophils might have distinct features.

### *Cxcr2-/-* neutrophils display altered phagocytic ability

**Figure 2:**
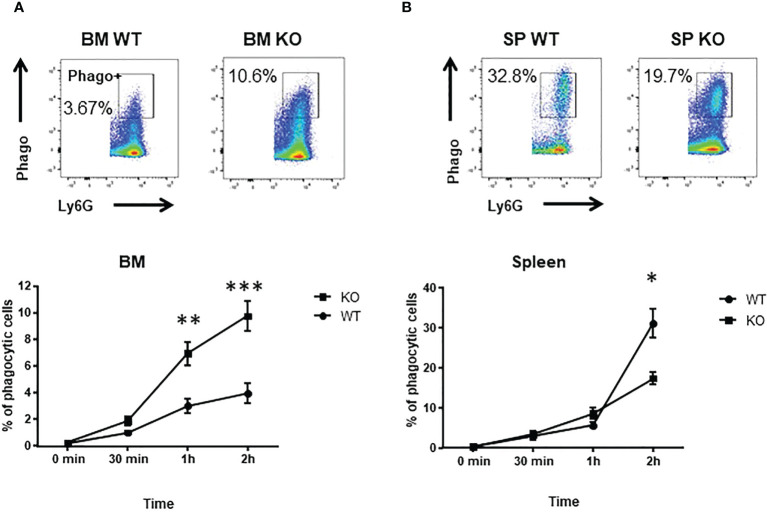
spleen *Cxcr2-/-* neutrophils have an impaired phagocytosis ability. **(A)** To measure phagocytosis, BM CD11b+ Ly6G+ neutrophils were incubated with Red *E*. coli Phrodo bioparticles at 37°C for 0, 30 min, 1h or 2h and analyzed by flow cytometry. Upper panel: Gating strategy to identify phagocytic neutrophils (Phago+) in the CD11b+ Ly6G+ fraction at 2h time. Lower panel: Percentage of phagocytic Ly6G+ neutrophils. **(B)** Same experiment with spleen neutrophils. Results are expressed as the percentage of phagocytic neutrophils in the CD11b+ Ly6G+ population and represent the mean ± SEM of at least 6 animals (Mann-Whitney test, *p < 0.05, **p < 0.01, ***p < 0.001).

Phagocytosis is one of the major function of neutrophils to eliminate infections. Thus, we analyzed the phagocytic abilities of BM and spleen neutrophils. Kinetics of phagocytosis showed that BM *Cxcr2-/-* neutrophils had an enhanced phagocytic ability compared to WT neutrophils (**Figure 2A**), whereas spleen *Cxcr2-/-* neutrophils were less phagocytic than spleen WT neutrophils (**Figure 2B**). We have also performed an additional analysis, by separating Ly6G^hi^ and Ly6G^lo^ neutrophils (**Supplemental Figure 5**). Similar results were obtained for Ly6G^hi^ neutrophils as for Ly6G+ neutrophils, which is an increase of phagocytosis for BM KO neutrophils and a decrease of phagocytosis for spleen KO neutrophils. Concerning Ly6G^lo^ neutrophils, they were less phagocytic than Ly6G^hi^ neutrophils, both in the spleen and BM. Moreover, BM KO Ly6G^lo^ were also more phagocytic than BM WT Ly6G^lo^ neutrophils. On the other hand, no difference was seen for spleen Ly6G^lo^ neutrophils between WT and KO animals. In addition, when using opsonized bioparticles (**Supplemental Figure 6**), we obtained the same trend of difference between WT and KO neutrophils as for non-opsonized particles.

**Figure 3:**
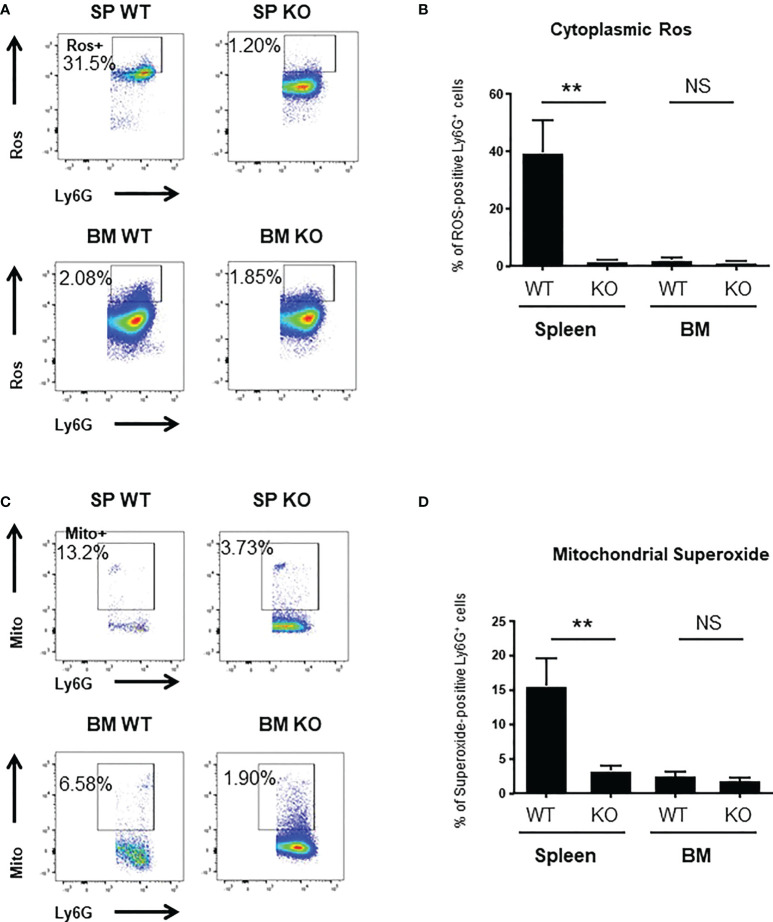
spleen *Cxcr2-/-* neutrophils display reduced ROS and superoxide levels. **(A)** Gating strategy to measure cytoplasmic Ros levels using CellRox Orange in CD11b+ Ly6G+ neutrophils. **(B)** ROS levels were quantified in spleen and BM CD11b+ Ly6G+ neutrophils using CellRox Orange probe. Results are expressed as the percentage of ROS-positive neutrophils in the CD11b+ Ly6G+ population and represent the mean ± SEM of at least 6 animals (Mann-Whitney test, NS: non-significant, **p < 0.01). **(C)** Gating strategy to measure Mitochondrial superoxide levels using Mitosox in CD11b+ Ly6G+ neutrophils. **(D)** Measure of the percentage of mitochondrial superoxide –positive CD11b+ Ly6G+ neutrophils using Mitosox reagent. Data represent the mean ± SEM of at least 6 animals (Mann-Whitney test, NS: non-significant, **p < 0.01).

**Figure 4:**
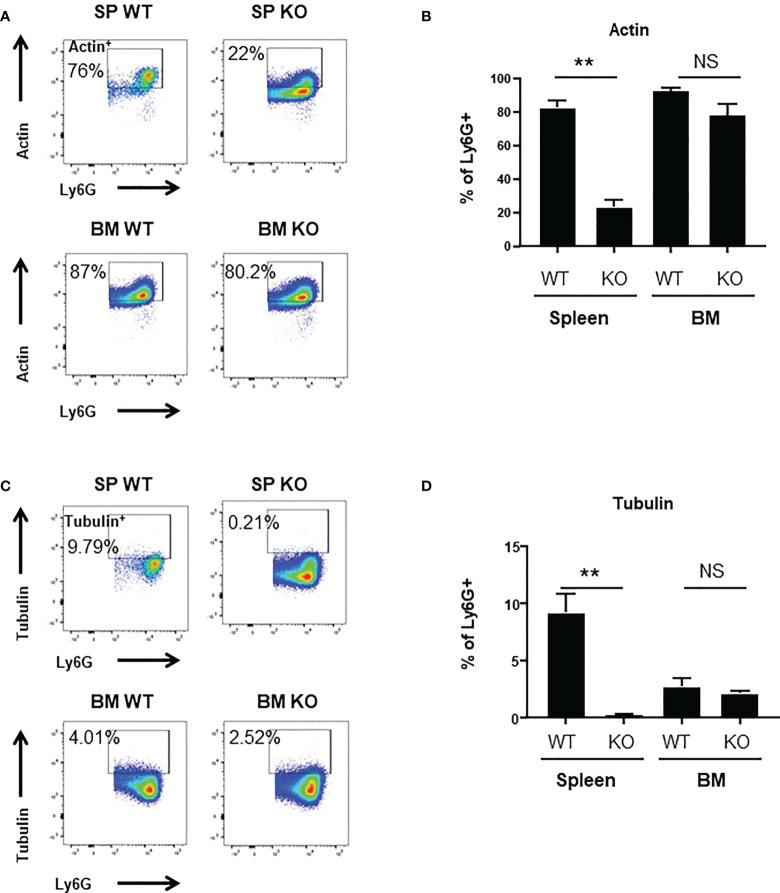
Decrease of Actin^+^ and Tubulin^+^ levels in spleen *Cxcr2-/-*. **(A)** Gating strategy to measure F-Actin levels (Actin^+^) in CD11b+ Ly6G+ neutrophils. **(B)** Percentage of CD11b+ Ly6G+ expressing Actin (mean ± SEM of at least 6 animals; Mann-Whitney test, NS: non-significant, **p < 0.01). **(C)** Gating strategy to measure α-Tubulin levels (Tubulin^+^) in CD11b+ Ly6G+ neutrophils. **(D)** Percentage of CD11b+ Ly6G+ expressing Tubulin. (mean ± SEM of at least 6 animals; Mann-Whitney test, NS: non-significant, **p < 0.01).

To better understand the mechanisms underlying these changes in phagocytic ability, we looked at ROS production and to actin-tubulin cytoskeleton organization of neutrophils, which are essential for phagocytosis (35). We observed a strong reduction in the production of cytoplasmic ROS (**Figures 3A, B**) and mitochondrial superoxide (**Figures 3C, D**) of spleen *Cxcr2-/-* neutrophils, whereas no difference could be measured between BM WT and *Cxcr2-/-* neutrophils. Spleen *Cxcr2-/-* neutrophils had also lower levels of F-Actin (**Figures 4A, B**) as well as tubulin (**Figures 4C, D**) compared to WT, but BM neutrophils were unaffected by *Cxcr2* impairment. These results of decreased levels of F-actin and tubulin were also confirmed, when comparing MFI values for F-actin and tubulin for the different types of neutrophils (**Supplemental Figure 7**). Altogether, these results could account for the lower phagocytic ability of spleen *Cxcr2-/-* neutrophils, which is in agreement with a lower maturity.

### Spleen *Cxcr2-/-* neutrophils have a higher viability

**Figure 5:**
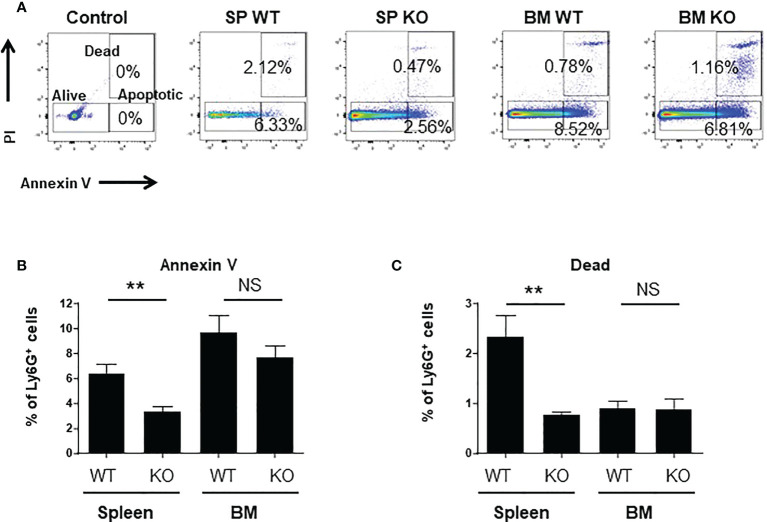
spleen *Cxcr2-/-* neutrophils exhibit a reduced apoptosis and mortality. **(A)** Gating strategy to identify alive, dead and apoptotic cells based on Annexin V and PI staining. Control plot corresponds to cells labelled with cell surface markers, but not with Annexin V and PI **(B)** Measure of the percentage of apoptotic neutrophils in the spleen and BM of WT and *Cxcr2-/-* animals by annexin V staining. **(C)** Same measure of dead cells by PI staining. Results are expressed as the percentage of CD11b+ Ly6G+ neutrophils and represent the mean ± SEM of 6 animals; Mann-Whitney test, NS: non-significant, **p < 0.01.

Neutrophil are short term living cells, so we wondered whether Cxcr2 could modulate their survival ability. We observed that the percentages of apoptotic or dead cells were reduced for spleen *Cxcr2-/-* neutrophils (**Figures 5A–C**, respectively), whereas it was similar for WT and *Cxcr2-/-* BM neutrophils. So, this suggests that the reduced phagocytic ability of spleen *Cxcr2-/-* neutrophils is not a consequence of an impaired survival. We have also measured apoptosis and death after a 2h incubation at 37°C, and obtained similar results (**Supplemental Figure 8**), which suggests that an increased apoptosis or death after 2h incubation does not affect the phagocytosis ability of neutrophils.

### Transcriptomic analysis confirms the impaired maturation of spleen *Cxcr2-/-* neutrophils

**Figure 6:**
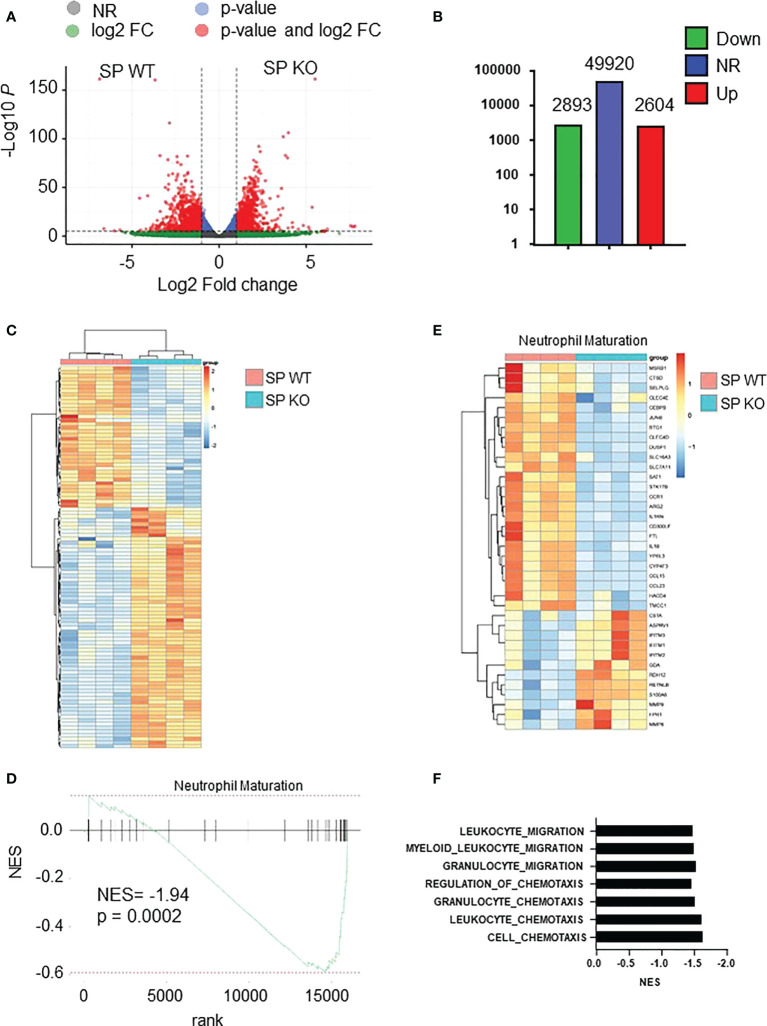
*Cxcr2-/-* neutrophils display decreased maturity at the transcriptomic level. **(A)** Volcano plot showing the global changes in RNA expression patterns for spleen neutrophils isolated from *Cxcr2-/-* (SP KO) versus WT (SP WT) animals. Data represent analysis of cpm estimates with a log of fold change of more than 1.5 fold and p< 0.05 of 4 animals per group. Grey dots: NR: non-regulated genes; Green dots: genes with a log of fold change of more than 1.5 fold; blue dots: genes with a p-value <0.05; red dots: genes with a log of fold change of more than 1.5 fold and p< 0.05. **(B)** Number of differentially regulated genes for the spleen neutrophils. Up: genes up-regulated in spleen isolated from *Cxcr2-/-* versus WT animals. Down: down-regulated genes. NR: non regulated genes. **(C)** Simplified Heatmap of spleen *Cxcr2-/-* versus WT neutrophils. **(D)** Normalized enrichment score (NES) after GSEA analysis of the transcriptome of spleen neutrophils isolated from *Cxcr2-/-* versus WT animals according to Neutrophil maturation of Xie et al. (36). **(E)** Heatmap of the significantly regulated genes (p<0.05) of Neutrophil maturation signature. **(F)** Cluster of chemotaxis and migration GSEA analysis from *Cxcr2-/-* versus WT animals according to Biologic process GO: Cell-chemotaxis (NES= -1.64; q= 0.0002), Leukocyte-chemotaxis (NES= -1.62; q= 0.001), Granulocyte chemotaxis (NES= -1.53; q= 0.029), Regulation of Chemotaxis (NES = -1.46; q = 0.013), Granulocyte migration (NES= -1.54; q=0.025), Myeloid-leukocyte-migration (NES= -1.50; q= 0.012), leukocyte-migration (NES = -1.48; q= 0.003).

We next focused on spleen neutrophils to decipher whether and how Cxcr2 deficiency impacts their molecular identity. RNAseq analyses of WT and *Cxcr2-/-* spleen neutrophils showed that more than 2,500 transcripts were up-regulated and about the same number down-regulated in *Cxcr2-/-* spleen neutrophils (**Figures 6A–C**). We then focused on their maturation using the neutrophil maturation signature reported by Xie and collaborators (36). GSEA analysis confirmed that spleen *Cxcr2-/-* were less mature than their WT counterparts (**Figures 6D, E**), with down regulation of genes encoding Cathepsin D (Ctsd), JunB or IL-1β involved in Netosis (2), Arginase-2 (Arg2) controlling extra-urea cycle arginine metabolism and nitric oxide synthesis (12), C-type lectin receptor Clec4d crucial for bacteria elimination (37) or Selplg (CD162), important for the rolling of neutrophils (38).

In addition, there was a cluster of GSEA Biological process (BP) signatures showing a down-regulation of the chemotaxis and migration of spleen *Cxcr2-/-* neutrophils (**Figure 6F**), suggesting a decrease in the migration ability of these neutrophils.

### ERK and p38 MAPK pathways are down-regulated in spleen *Cxcr2-/-* neutrophils

**Figure 7:**
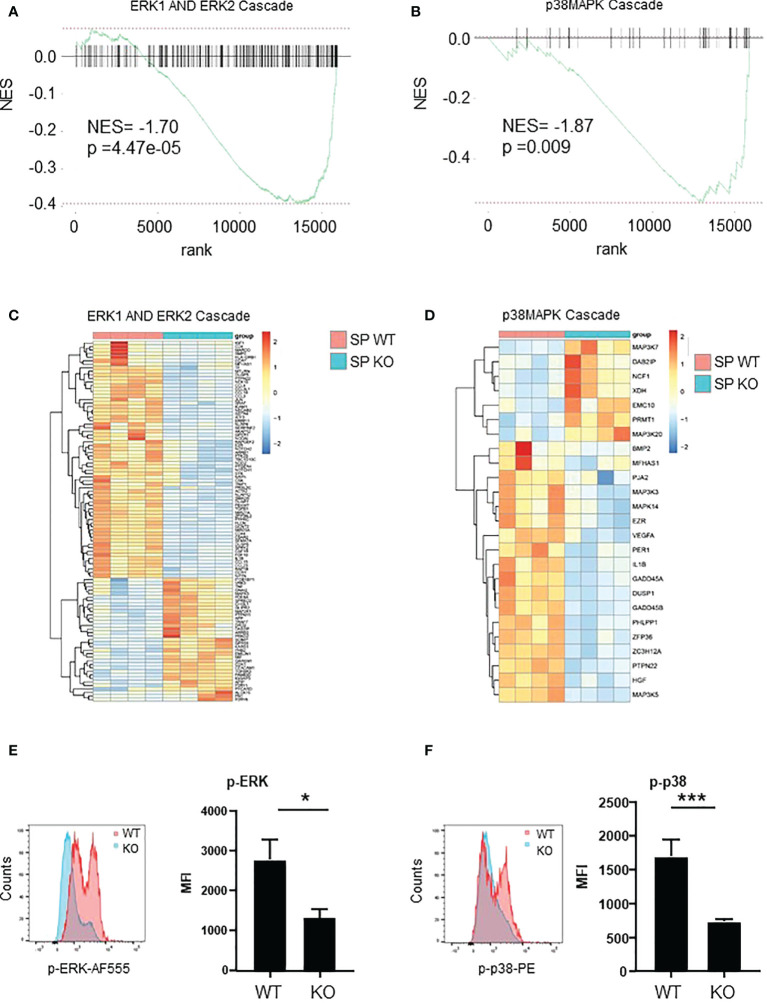
ERK and p38 pathways are down-regulated in Spleen *Cxcr2-/-* neutrophils. **(A)** NES after GSEA analysis of the transcriptome of spleen neutrophils isolated from *Cxcr2-/-* versus WT animals according to ERK1 and ERK2 cascade signature. **(B)** Same analysis for p38-MAPK signature. **(C)** Heatmap of the significantly regulated genes (p<0.05) of GO-BP ERK1 and ER2 cascade signature. **(D)** Heatmap of the significantly regulated genes (p < 0.05) of GO-BP p38 MAPK cascade signature. **(E)** Phospho-p42/44 MAPK ERK1/2 (Thr 202/Tyr 204) was analyzed by flow cytometry in spleen CD11b+ Ly6G+ neutrophils. Left panel: representative Mean of Fluorescence (MFI) of WT (red line) and KO (blue line) Ly6G+ neutrophils. Results are expressed as MFI of phospho-ERK positive neutrophils in the CD11b+ Ly6G+ population and represent the mean ± SEM of at least 6 animals (Mann-Whitney test, *p < 0.05). **(F)** Phosphorylation of p38MAPK on Thr180/Tyr182 was analyzed by flow cytometry in the same conditions (Mann-Whitney test, ***p < 0.001).

To look at the signaling that were affected in spleen *Cxcr2-/-* neutrophils, we first focused on ERK and p38 MAPK signaling, which are known to modulate the migration and adhesion of neutrophils (39, 40). RNAseq data showed that both ERK1 and ERK2 cascade (**Figures 7A, C**), as well as p38MAPK cascade (**Figures 7B, D**), were down regulated in spleen *Cxcr2-/-* neutrophils. Concerning ERK1/2 cascade, there is in particular a down-regulation of the AP-1 Transcription Factor Jun, of the Cyclic AMP-Dependent Transcription Factor Atf-3, IL1β, growth factors such as TGFβ1 and IGF1, multiple chemokines such as Ccl3, 4, 15, 18, 23 and of a number of kinases such as Ptk2b (Protein Tyrosine Kinase 2 Beta), the tyrosine-protein kinase Syk, Csk (C-Terminal Src Kinase) or the serine/threonine protein kinase BRAF (**Figure 7C**). For p38MAPK cascade, we observed in particular a down regulation of Mitogen-Activated Protein Kinase 14 (Mapk14), Mitogen-Activated Protein Kinase Kinase Kinase 3 (Map3k3), Map3k5, and cytokines such as IL1β, HGF.

To confirm these results, we measured by flow cytometry the intracellular content of Phospho-p42/44 MAPK ERK1/2 and phospho-p38 MAPK (Thr 180/Tyr 182). The mean of fluorescence (MFI) of p-ERK1/2 and p-p38 were down-regulated by about 50% in spleen *Cxcr2-/-* neutrophils compared to WT neutrophils (**Figures 7E, F**, respectively) confirming the alterations of these pathways.

### Several pathways are impaired in spleen *Cxcr2-/-* neutrophils

**Figure 8:**
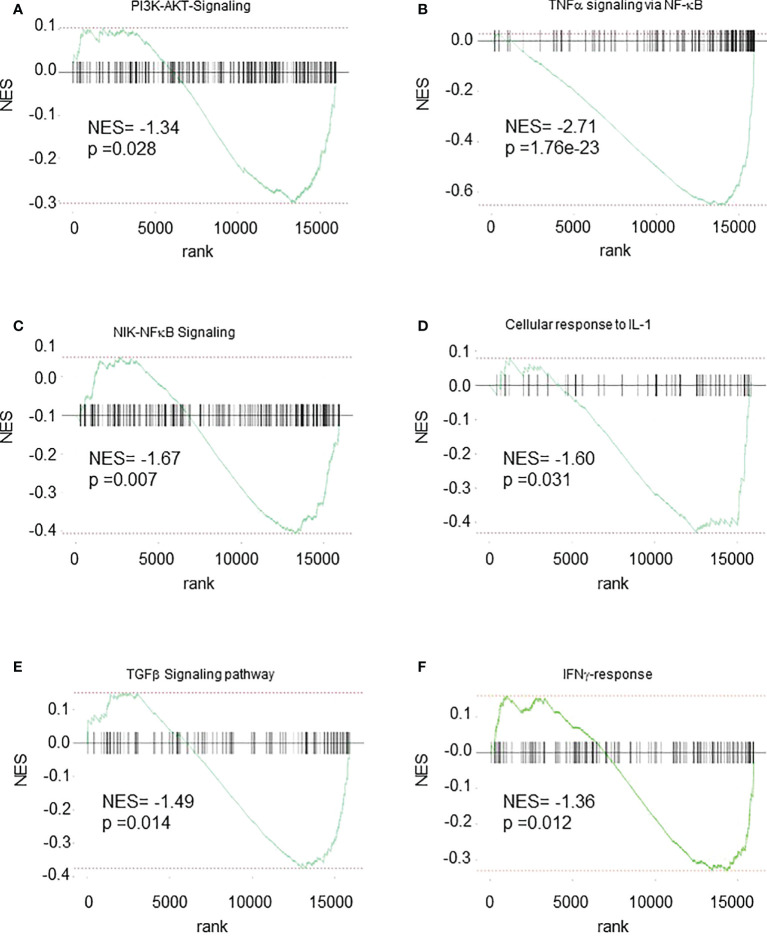
Multiple pathways are down-regulated in Spleen *Cxcr2-/-* neutrophils. GSEA analysis of the following signatures: **(A)** WIKI-PI3K-AKT signaling. **(B)** Hallmark-TNFα signaling *via* NFκB. **(C)** GO-BP NIK-NFκB Signaling **(D)** GO-BP Cellular response to IL-1 **(E)** WP-TGFβ signaling pathway **(F)** Hallmark IFNγ-response.

To further investigate the mechanisms involved in the changes observed in spleen *Cxcr2-/-* neutrophils, we explored the main pathways that were affected at the transcriptomic level. GSEA analysis showed a reduction of PI3K-AKT signaling (**Figure 8A**), TNFα signaling *via* NFκB (**Figure 8B**), NIK-NFκB signaling (**Figure 8C**), as well of the cellular response to IL-1 (**Figure 8D**) and TGFβ signaling (**Figure 8E**) and signaling by IFNγ (**Figure 8F**) signatures in spleen *Cxcr2-/-* neutrophils compared to WT neutrophils. Among the genes involved in PI3K-AKT pathway, we observed a down-regulation of Phosphatidylinositol-4,5-Bisphosphate 3-Kinase Catalytic Subunit Alpha, Delta and Gamma (Pik3ca, Pik3cd and Pik3cg), and Phosphoinositide-3-Kinase Regulatory Subunit 1, 2 and 5 (Pik3r1, Pik3r2, Pik3r5), Janus Kinase 1 (Jak1), Glycogen Synthase Kinase 3 Beta (GSK3b), and Kras, which are crucial for this pathway.

NF-κB pathway was also impaired with in particular down-regulation of genes such as NF-κB Subunits p52 (Nfkb2), Relb, C-Rel (Rel), TNF Alpha Induced Protein 2 and 3 (Tnfaip2 and Tnfaip3), Tnfaip3 Interacting Protein 1 (Tnip1), TNF Receptor-Associated Factor 1, 3 and 5 (Traf1, Traf3, Traf5) or TNF Receptor Superfamily Member 10b (Tnfrsf10b).

Upstream of NF-κB activation, cellular response to IL-1 was also altered, with a down-regulation of IL-1 itself, but also of Interleukin 1 Receptor Type 1 (Il1r1). Two other pathways were also linked to PI3K/Akt, ERK, p38 and NF-κB signaling, namely TGFβ and IFNγ signaling. TGFβ signaling was impaired with a decrease in Transforming Growth Factor Beta 1 (Tgfb1), Transforming Growth Factor Beta Receptor 1 and 2 (Tgfbr1 and Tgfbr2), and Smad2, 3, and 4. IFNγ signaling is also acting through ERK and MAPK and PI3K signaling and exhibits in particular a down-regulation of Janus kinase 2 (Jak2), suppressor of cytokine signaling protein 1n and 3 (Socs1, Socs3), Signal transducer and activator of transcription 4 (Stat4), Intercellular adhesion molecule-1 (ICAM-1) and Integrin Subunit Beta 7 (ITGB7).

## Discussion

Although the role of neutrophils in general inflammation but also tumor microenvironment inflammation is growing, the factors controlling their function are still not completely identified. In particular, the involvement of Cxcr2 in neutrophil physiology and features, as well as its impact on signalization in neutrophils is still a matter of debate. In this study, we report that impairment of *Cxcr2* leads to a modest increase of the percentage of neutrophils in the BM, but a strong one in the spleen, in agreement with previous studies (29). We also show that Cxcr2 differentially affects the maturation state of neutrophils in the spleen and the BM. More precisely, based on the maturation markers Ly6G (32) and Cd101 (8) expression, *Cxcr2* deletion led an increase of Ly6G^lo^ or CD101-immature neutrophils in the spleen, but to a decrease of immature neutrophils in the BM. The presence of resident immature neutrophils in the spleen is thought to serve as a reservoir of neutrophils, which will undergo a rapid proliferation and mobilization in case of infection to increase the number of active mature neutrophils (18, 32). Spleen is also a site of accumulation and destruction of neutrophils in human (41), so the presence of a high number of immature neutrophils could correspond to both resident and recruited neutrophils. On the other hand, considering that the BM is the main site of production of neutrophils, this would suggest that Cxcr2 impairment does not alter the maturation process on neutrophils in the BM, or that there could be a retention of mature neutrophils in BM. This latter hypothesis is unlikely, as we did not see any difference in the percentage Cxcr4+ neutrophils in the BM. Cxcr4 is indeed crucial for the retention of neutrophils in the BM (17), even though other studies have shown that the CXCR4 antagonist plerixafor did not mobilize neutrophils from the BM, but rather enhanced the release of neutrophils in the circulation from the marginated pool present in the lung (42). In contrast, the percentage of Cxcr4+ neutrophils was increased 2-fold in the spleen, which could account for several features: this could be the sign of more immature, since Cxcr4 expression is high in proliferating immature neutrophils (8). However, high levels of Cxcr4 can also be seen in senescent or aging neutrophils (17, 43). To clarify this point, we analyzed the presence of aged CD62L^lo^ – CXCR4^hi^ neutrophils as defined previously (33). This shows that there is an increase of aged CD62L^lo^ – CXCR4^hi^ neutrophils in the spleen of KO animals, but no change in BM. In addition, we also observed that the percentage of apoptotic or dead neutrophils was reduced among spleen *Cxcr2-/-* neutrophils compared to WT, whereas no difference was seen in BM. Overall, our data suggest that spleen *Cxcr2-/-* neutrophils are distinct from BM neutrophils, as they are healthier and have more immature features (Ly6Glo Cd101- Cxcr4+) than their WT counterparts.

To go further in the understanding of the effects of Cxcr2 impairment, we have observed that spleen *Cxcr2-/-* neutrophils exhibited a reduced phagocytic ability than WT neutrophils. On the other hand, BM *Cxcr2-/-* neutrophils had a higher phagocytic ability than the WT. This difference between spleen and BM neutrophils might be explained by their difference of maturation mentioned above. This is also true for basal phagocytic ability of WT neutrophils, which is higher in the spleen than in the BM. This might be due to a higher proportion of mature CD101+ neutrophils in WT spleen compared to WT BM (**Figure 1F**), as we have also shown that Ly6G^hi^ neutrophils had a better phagocytic ability than Ly6G^lo^ neutrophils (**Supplemental Figure 5**).

To explore the difference in phagocytic ability of WT and *Cxcr2-/-* neutrophils more thoroughly, we looked at ROS production and actin and tubulin cytoskeleton, which are key elements in phagocytosis (35, 44). The percentage of cells with high cytoplasmic ROS, mitochondrial superoxide, F-actin and α-tubulin was reduced among spleen *Cxcr2-/-* neutrophils compared to WT, but was not modified in BM neutrophils. This reduction could explain part of the reduced phagocytic ability of spleen *Cxcr2-/-* neutrophils, although one can also notice that decreased phagocytic ability of WT BM neutrophils compared to WT spleen neutrophils is not correlated to their actin levels, suggesting that other parameters might be involved. It is interesting to notice that aged neutrophils are resistant to infections and display also a reduction of actin levels (43). Moreover, it has also been shown that in some cases, immature neutrophils could produce less ROS (8).

It was essential to analyze the mechanisms underlying the differences between spleen *Cxcr2-/-* and WT neutrophils. RNAseq analysis of both types of isolated neutrophils confirmed the defect in maturation of spleen *Cxcr2-/-* neutrophils, with in particular a down regulation of genes involved in Netosis (Cathepsin D, JunB or IL-1β) (45), of Arginase-2 controlling extra-urea cycle arginine metabolism and nitric oxide synthesis, of Clec4d implicated in bacteria elimination (46) or Selplg (CD162), essential for the rolling of neutrophils (47). Several GO pathways of migration and chemotaxis constituted a down-regulated cluster, suggesting also an impairment of spleen *Cxcr2-/-* chemotaxis. This could explain an accumulation of neutrophils in the spleen, with a weak ability to migrate to other tissues. Earlier studies have shown that in inflammatory conditions, Cxcr2 was essential for the recruitment of neutrophils (48).

In terms of signaling, we report a down regulation of ERK and p38 MAPK pathways, both at the transcriptomic level, but also when assessing the phosphorylation of ERK and p38 at the protein level. The ERK and p38 MAP kinases are strongly stimulated in neutrophils upon activation by G-protein coupled receptors agonists. Moreover, p38 MAPK is critical for the release of primary and secondary granules, but not that of secretory vesicles by neutrophils (49). p38 inhibition has been shown to delay apoptosis of neutrophils (50), which is in agreement of the reduced rate of apoptosis that we observed in spleen *Cxcr2-/-* neutrophils. Moreover, p38 MAPK promotes chemotaxis towards fMLP by interfering with GRK2-mediated desensitization, whereas ERK MAPK is inhibiting it (51, 52). However, the role of ERK MAPK pathway in neutrophil functions remains unclear, due to contradictory studies (37).

In addition, PI3K-Akt, TNFα signaling and NF-κB signaling, were also altered. TNFα is essential to trigger neutrophil activation and phagocytic activity (53). It is interesting to note that in neutrophils, the production of ROS is dependent on PI3K and ERK (54). Moreover, PI3K, ERK and p38 are necessary for efficient phagocytosis (55). It has also been reported that PI3Kγ-/- neutrophils display a defect in migration towards fMLP, C5a, Cxcl8 or Ccl3 and respiratory burst upon activation by C5a or fMLP (56), but another study suggests that PI3K is not required for chemotaxis towards fMLP (51).

Cellular response to IL-1 was also reduced. IL1α and IL1β are very potent mediators of inflammation response, but their role in neutrophils remains poorly understood. Their main effect on neutrophils could be to increase their survival (37).

We also observed a down-regulation of TGFβ and IFNγ pathways. In the steady state situation, the role of TGFβ on neutrophil function is poorly understood. However, in cancer context, TGFβ is responsible for promoting the generation of pro-tumoral type N2 neutrophils (57), whereas type IFNβ and IFNγ might favor anti-tumoral type N2 neutrophils (58, 59). In non-cancerous situation, IFNγ has also been shown to enhance, or prime, increased ROS production in combination with a secondary stimulus and to promote phagocytosis (60), which could account for the decrease in ROS and phagocytosis that we observed in spleen *Cxcr2-/-* neutrophils. As treatment of PMNs with IFNγ increases the production of TNFα and IL-1β (61), this could also account for the down-regulation of TNFα and IL1β signaling that we report.

In conclusion, this work highlights the multiple roles played by the chemokine receptor Cxcr2 in neutrophils and reinforces the importance of localization of neutrophils in terms of action and features. The identification of the pathways that are dependent on Cxcr2 and their further investigation will be essential to understand the roles of Cxcr2 in neutrophils in the steady state or inflammatory situation but also in the tumoral context.

## Data availability statement

The datasets presented in this study can be found in online repositories. The names of the repository/repositories and accession number(s) can be found in the article.

## Ethics statement

The animal study was reviewed and approved by Ministère de l’enseignement supérieur, de la recherche et de l’innovation.

## Author contributions

PD, BG, and ER and have contributed to the investigation. KB participated to writing review and editing. GL was in charge of the conceptualization, investigation and supervision of the project, funding acquisition and writing original draft preparation. All authors have read, revised and agreed to the published version of the manuscript.

## Funding

This work was supported by la Ligue contre le Cancer to GL.

## Acknowledgments

We acknowledge the PCEA, RAM, MRI facilities in Montpellier. We are grateful to Institut du cerveau et de la moëlle épinière in Paris for RNAseq experiments. We thank Vincent Rondeau and Marie-Laure Aknin for useful advices in protocols.

## Conflict of interest

The authors declare that the research was conducted in the absence of any commercial or financial relationships that could be construed as a potential conflict of interest.

## Publisher’s note

All claims expressed in this article are solely those of the authors and do not necessarily represent those of their affiliated organizations, or those of the publisher, the editors and the reviewers. Any product that may be evaluated in this article, or claim that may be made by its manufacturer, is not guaranteed or endorsed by the publisher.
