# Supplementary material for: CXCR2 intrinsically drives the maturation and function of neutrophils in mice
Source: Front Immunol. 2022 Oct 13;13:1005551. doi: 10.3389/fimmu.2022.1005551 (PMC9606682; doi:10.3389/fimmu.2022.1005551)
Supplement: Supplementary file 1 [file DataSheet_1.pdf]

Supplementary Material

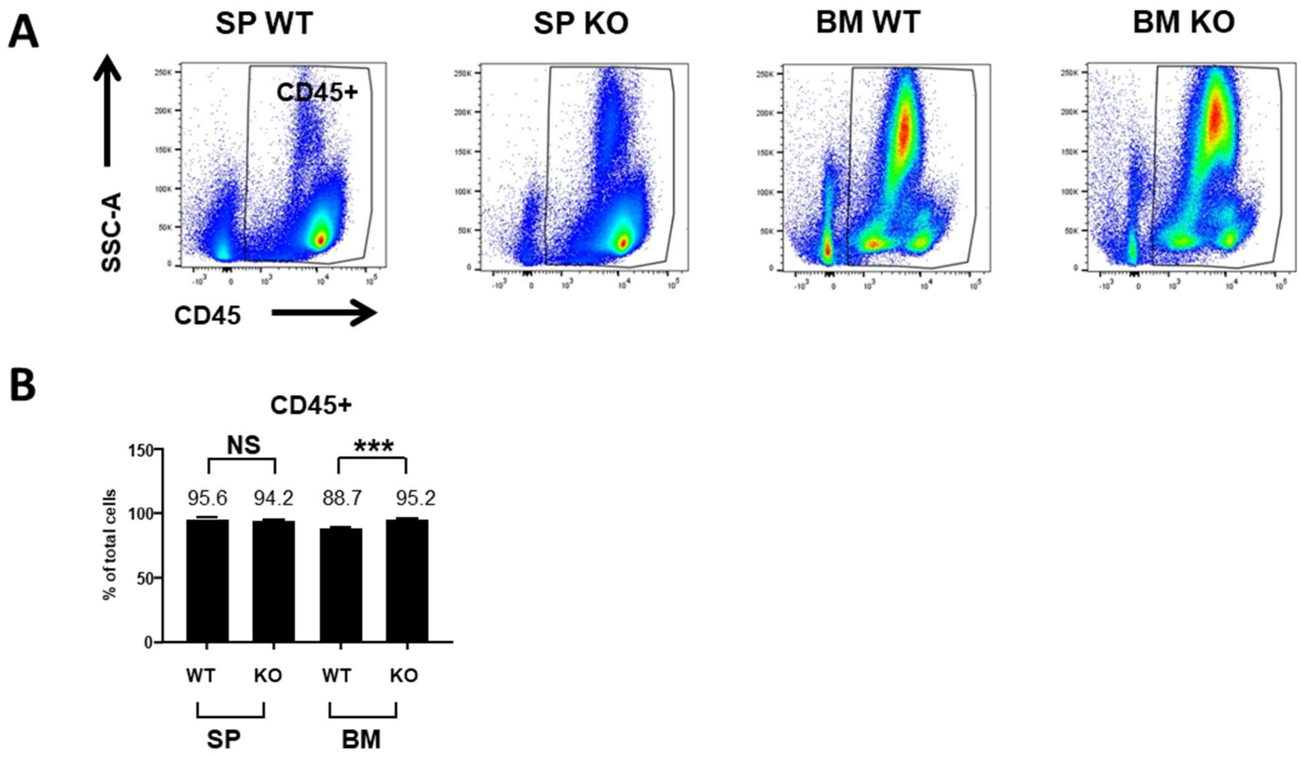

Supplemental Fig. 1: The percentage of CD45+ in WT and KO spleens is not modified.
A. Representative dot plots of the gating strategy of CD45+ cells in WT and *Cxcr2-/-* bone marrow (BM) and spleen (SP). **B.** Quantification of the percentage of CD45+ cells among total cells. Data represent the mean ± SEM of at least 6 animals (Mann-Whitney test, NS: non-significant, *** $p<0.001$).

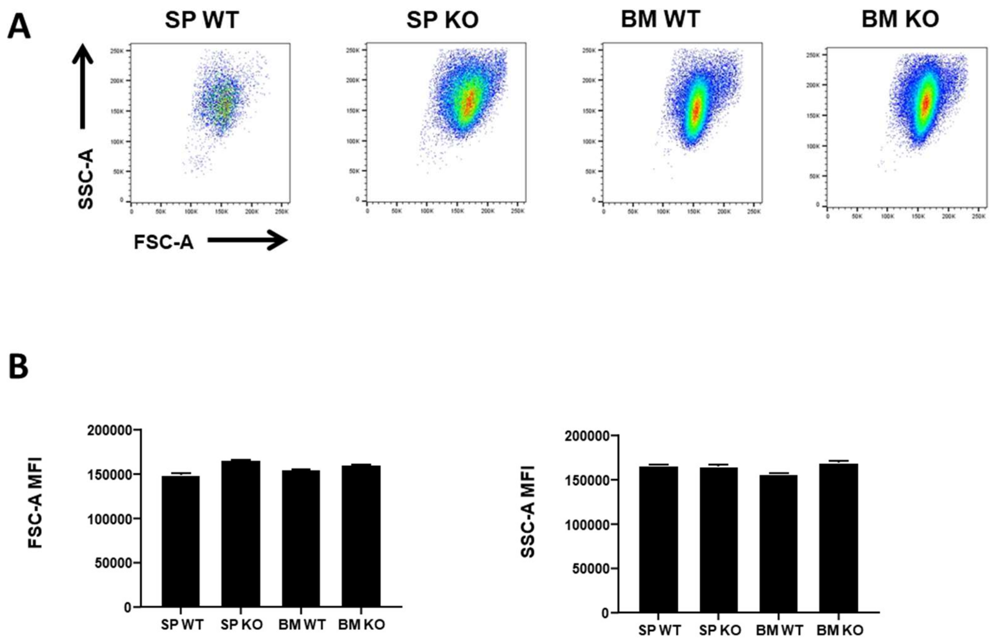

Supplemental Fig. 2: FSC and SSC gating of CD11b+ Ly6G+ neutrophils.
A. Representative dot plots of the gating of CD45+ CD11b+ Ly6G+ cells in WT and *Cxcr2-/-* bone marrow (BM) and spleen (SP). **B.** Mean of fluorescence of FSC-A and SSC-A CD45+ CD11b+ Ly6G+ neutrophils. Data represent the mean ± SEM of 3 animals (Mann-Whitney test, NS: non-significant).

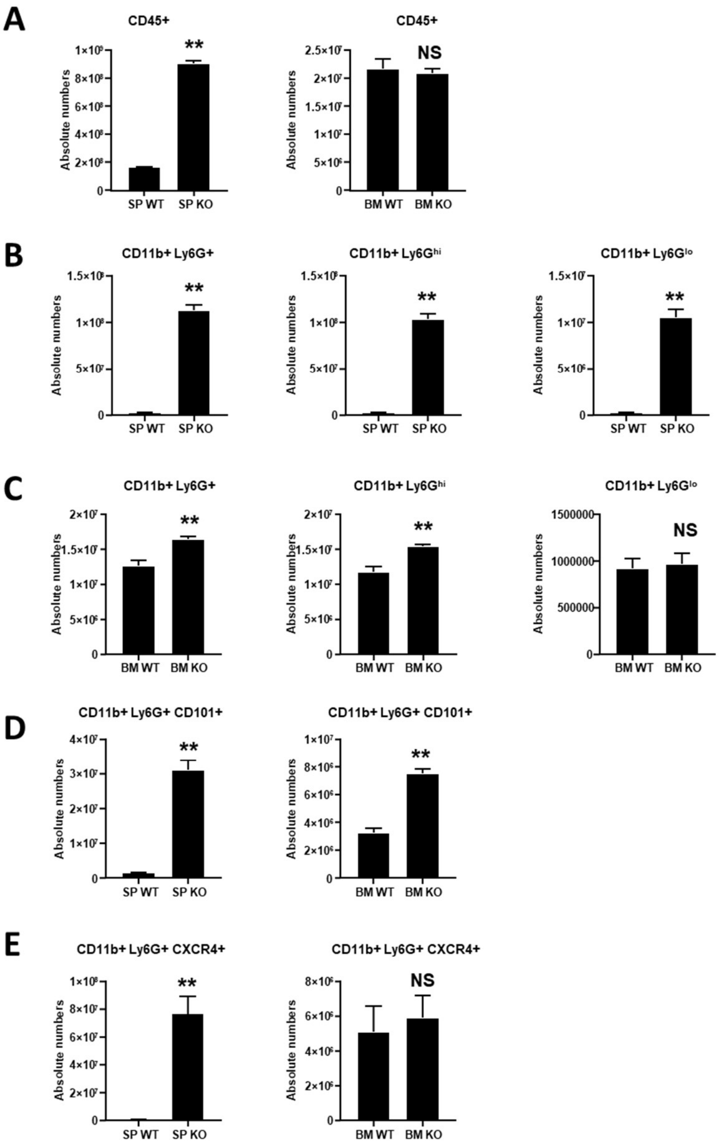

Supplemental Fig. 3: Absolute numbers of the different populations.
A. Absolute numbers of CD45+ cells in WT and *Cxcr2-/-* spleen (SP, left panel) and bone marrow (BM, right panel). Data represent the mean ± SEM of at least 6 animals (Mann-Whitney test, NS: non-significant, ** p<0.01). **B.** Same quantification for CD45+ CD11b+ Ly6G+ cells (left panel), CD45+ CD11b+ Ly6Ghi (middle panel) and CD45+ CD11b+ Ly6Glo (right panel) in the spleen (Mann-Whitney test, ** p<0.01). **C.** CD45+ CD11b+ Ly6G+ cells (left panel), CD45+ CD11b+ $Ly6G^{hi}$ (middle panel) and CD45+ CD11b+ $Ly6G^{lo}$ (right panel) in the BM (Mann-Whitney test, NS: non significant, ** p<0.01) **D.** CD45+ CD11b+ Ly6G+ CD101+ cells in the spleen (SP, left panel) and bone marrow (BM, right panel) (Mann-Whitney test, ** p<0.01). **E.** CD45+ CD11b+ Ly6G+ CXCR4+ cells in the spleen (SP, left panel) and bone marrow (BM, right panel) (Mann-Whitney test, NS, ** p<0.01).

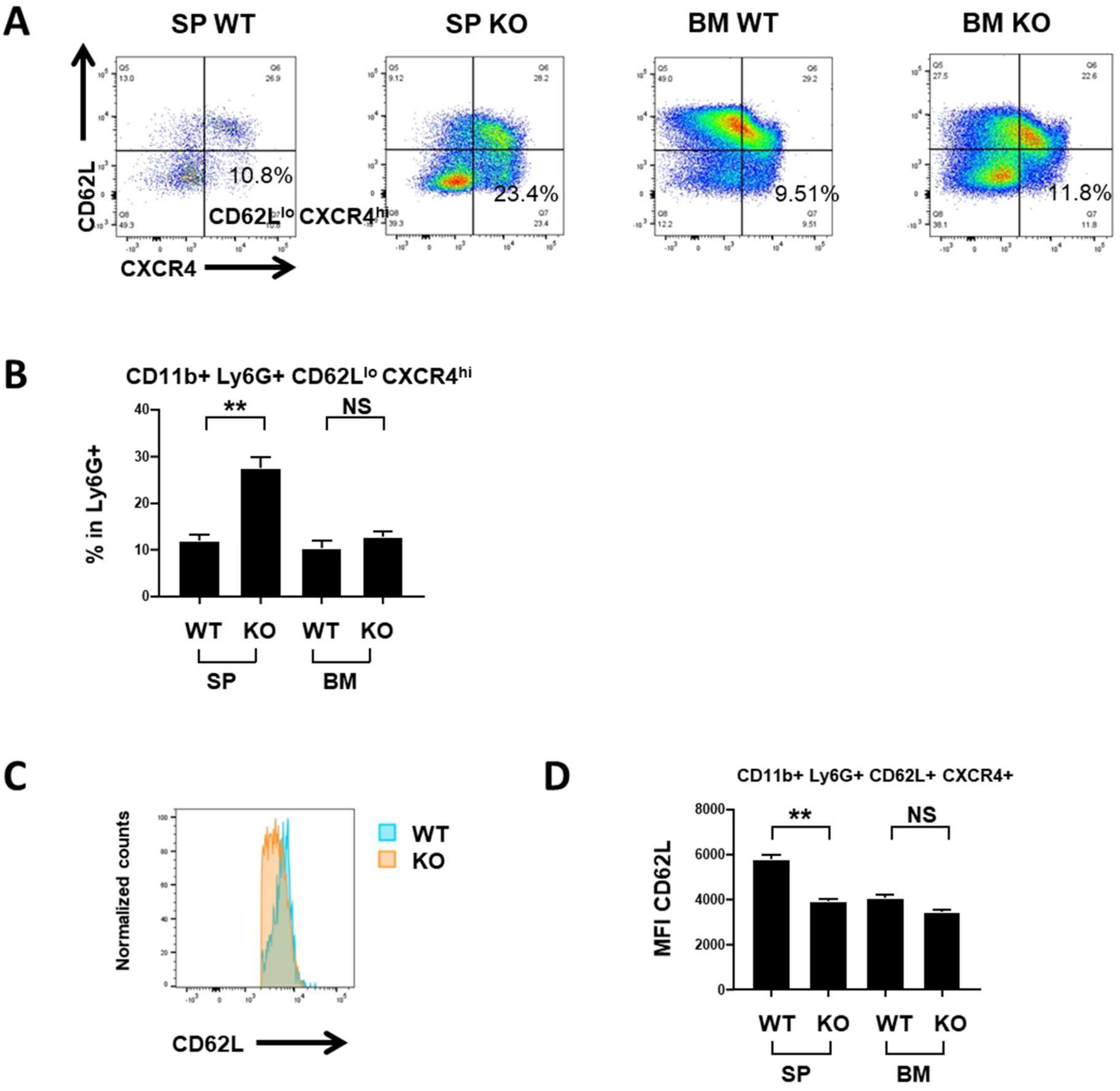

Supplemental Fig. 4: Increased percentage of CD62L^{lo} –CXCR4^{hi} neutrophils in the CD45+ CD11b+ Ly6G+ fraction of Cxcr2-/- spleen.

A. Gating strategy to identify CD62L^{lo} –CXCR4^{hi} neutrophils (Lower right quarter) in the CD45+ CD11b+ Ly6G+ fraction. **B.** Percentage of aged CD62L^{lo} –CXCR4^{hi} neutrophils in the CD45+ CD11b+ Ly6G+ fraction. Data represent the mean ± SEM of at least 4 animals (Mann-Whitney test, NS: non-significant, ** p<0.01). **C.** Representative histogram CD62L distribution in the CXCR4+ CD62L+ population of neutrophils (from right upper quadrant of Supp. Fig 4A) for spleen WT and spleen KO animals. **D.** Mean of fluorescence of CD62L in CD45+ CD11b+ Ly6G+ CXCR4+ CD62L+ neutrophils. Data represent the mean ± SEM of at least 4 animals (Mann-Whitney test, NS: non-significant, ** p<0.01).

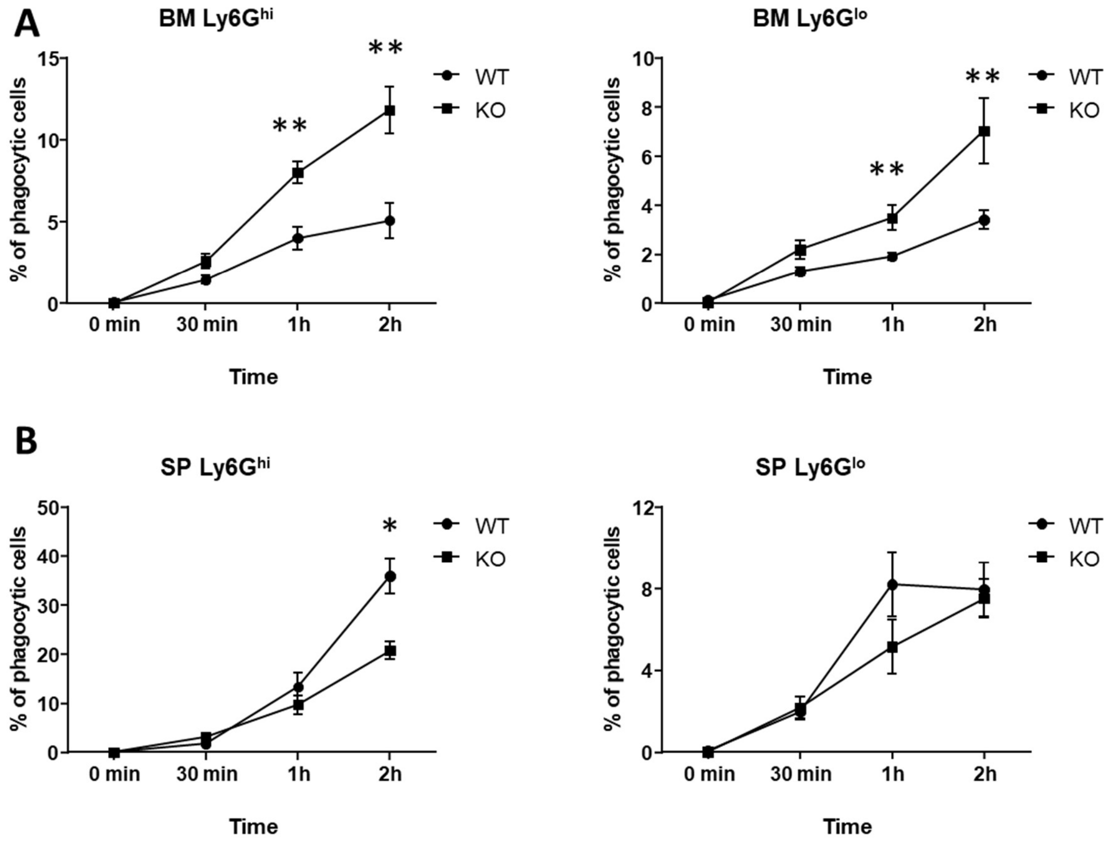

Supplemental Fig. 5: Phagocytic ability of Ly6G^{hi} and Ly6G^{lo} neutrophils.
A. To measure phagocytosis, BM neutrophils were incubated with Red E. coli Phrodo bioparticles at 37°C for 0, 30 min, 1h or 2h and analyzed by flow cytometry. Left panel: Percentage of phagocytic Ly6G^{hi} BM neutrophils. Right panel: Percentage of phagocytic Ly6G^{lo} BM neutrophils **B.** Same experiment with spleen Ly6G^{hi} and Ly6G^{lo} neutrophils. Results are expressed as the percentage of phagocytic neutrophils in the CD11b+ Ly6G+ population and represent the mean ± SEM of at least 6 animals (Mann-Whitney test, * $p<0.05$, ** $p<0.01$).

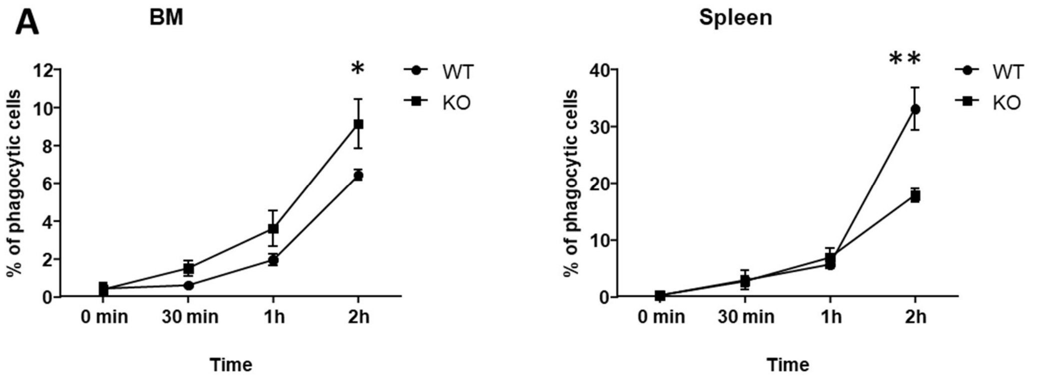

Supplemental Fig. 6: Phagocytic ability of opsonized particles by neutrophils.
A. To measure phagocytosis, CD11b+ Ly6G+ neutrophils were incubated with opsonized Red E. coli Phrodo bioparticles at 37°C for 0, 30 min, 1h or 2h and analyzed by flow cytometry. **B.** Same experiment with spleen Ly6G$^+$ neutrophils. Results are expressed as the percentage of phagocytic neutrophils in the CD11b+ Ly6G+ population and represent the mean ± SEM of at least 6 animals (Mann-Whitney test, * $p<0.05$, ** $p<0.01$).

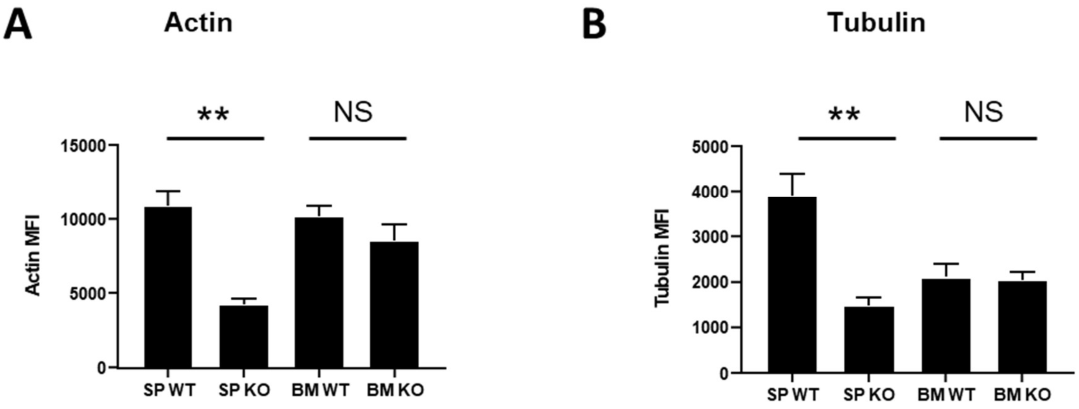

Supplemental Fig. 7: Actin and Tubulin mean of Fluorescence (MFI) of CD11b+ Ly6G+ neutrophils.

A. Mean of fluorescence of Actin for CD45+ CD11b+ Ly6G+ neutrophils. **B.** MFI of tubulin for CD45+ CD11b+ Ly6G+ neutrophils Data represent the mean ± SEM of 6 animals (Mann-Whitney test, NS: non-significant, ** p<0.01).

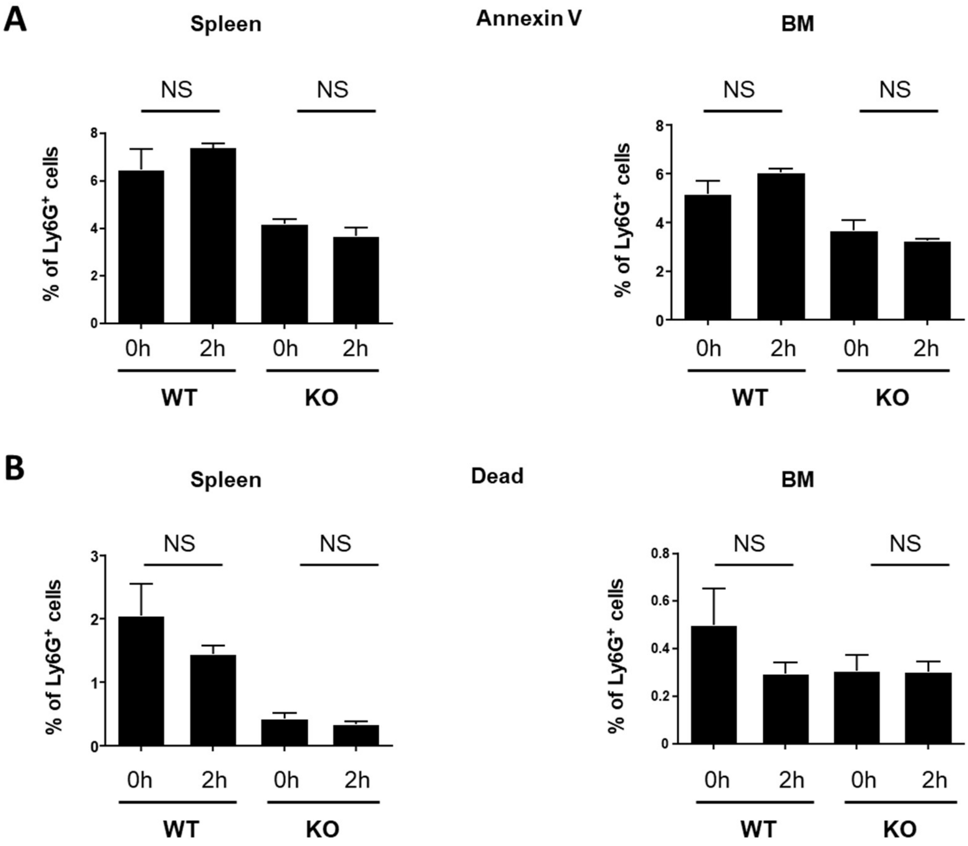

Supplemental Fig. 8: Effect of 2h incubation on apoptosis and death
A. Measure of the percentage of apoptotic neutrophils in the spleen and BM of WT and Cxcr2-/- animals by annexin V staining after no incubation (0h) or a 2h incubation at 37°C. **B.** Same measure of dead cells by PI staining. Results are expressed as the percentage of CD11b+ Ly6G+ neutrophils and represent the mean ± SEM of 3 animals; Mann-Whitney test, NS: non-significant).

.

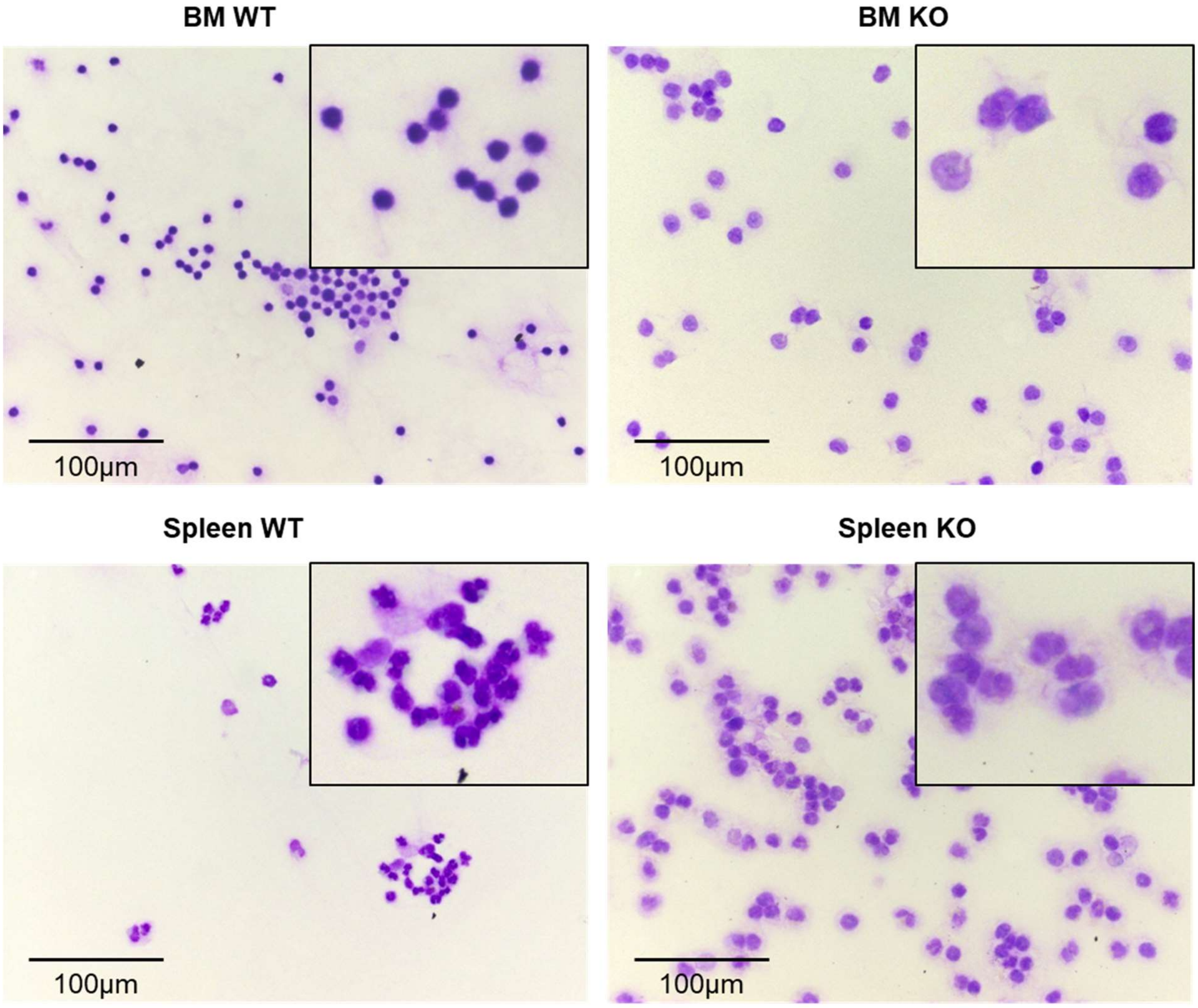

Supplemental Fig. 9: Morphology of neutrophils.
Representative pictures of purified CD45+ CD11b+ Ly6G+ neutrophils from WT BM (upper left panel), KO BM (upper right panel), WT spleen (lower left panel) and KO spleen (lower right panel) stained with Giemsa. Main image at a magnification of 400X. Incrusted images correspond to a zoom of these images.
